# Bacterial secreted effectors and caspase-3 interactions

**DOI:** 10.1111/cmi.12368

**Published:** 2014-10-30

**Authors:** Daniel M Wall, Beth A McCormick

**Affiliations:** 1 Institute of Infection, Immunity and Inflammation, University of GlasgowGlasgow, G12 8QQ, UK; 2 University of Massachusetts Medical SchoolWorcester, MA, USA

## Abstract

Apoptosis is a critical process that intrinsically links organism survival to its ability to induce controlled death. Thus, functional apoptosis allows organisms to remove perceived threats to their survival by targeting those cells that it determines pose a direct risk. Central to this process are apoptotic caspases, enzymes that form a signalling cascade, converting danger signals via initiator caspases into activation of the executioner caspase, caspase-3. This enzyme begins disassembly of the cell by activating DNA degrading enzymes and degrading the cellular architecture. Interaction of pathogenic bacteria with caspases, and in particular, caspase-3, can therefore impact both host cell and bacterial survival. With roles outside cell death such as cell differentiation, control of signalling pathways and immunomodulation also being described for caspase-3, bacterial interactions with caspase-3 may be of far more significance in infection than previously recognized. In this review, we highlight the ways in which bacterial pathogens have evolved to subvert caspase-3 both through effector proteins that directly interact with the enzyme or by modulating pathways that influence its activation and activity.

## Apoptosis – non-inflammatory cell death

Apoptosis was first discovered over forty years ago and has since been studied in intricate detail, generating a complex web of interactions that define this process (Kerr *et al.*, 1972). Once initiated, the process of apoptosis proceeds rapidly with cell shrinkage, nuclear condensation, DNA fragmentation and the formation of apoptotic bodies, which eventually dissociate from the cell and become engulfed and destroyed by circulating phagocytes. Apoptosis has been linked to several disease states with increases in apoptosis leading to degenerative disease [i.e. Alzheimer's, Parkinson's, amyotrophic lateral sclerosis (Pasinelli and Brown, 2006; da Costa and Checler, 2010; Tischner *et al.*, 2010; Crews *et al.*, 2011; Song *et al.*, 2011)] while decreases in, or mis-regulation of, apoptosis can lead to auto-immune disease [i.e. rheumatoid arthritis, systemic lupus erythematosus (Eguchi, 2001; Favaloro *et al.*, 2012)] or tumour development (Favaloro *et al.*, 2012). Apoptosis can be initiated from within or outside the host cell by stimuli including, microbial infection, oxidative stress, acquisition of tumorigenic potential, DNA damage or protein mis-folding, or simply when the cell has reached the end of its life cycle.

Once the process of apoptosis is induced, it engages a cascade of caspase enzyme activation, which is facilitated by upstream ‘initiator caspases’ that eventually results in controlled self-destruction of the cell caused by DNA fragmentation and cleavage of essential proteins by ‘executioner caspases’, namely caspases-3, -6 and -7 (Nuñez *et al*., 1998). Executioner caspase activation occurs in cells as both intrinsic and extrinsic signals deemed detrimental to the cell are acted on, and one of two distinct signalling cascades becomes activated. Executioner caspases then trigger DNases, and cleave essential cellular proteins, effectively destroying the cell from within and choreographing the cellular events in apoptosis leading to the cell being removed or phagocytosed by circulating immune cells. Despite apoptosis being a well-defined process owing to its essential role in cellular survival, the function it plays during infection is still unclear. Contrary to the long accepted dogma of apoptosis serving a protective role for the host, recent investigation into bacterial infections reveals apoptosis and apoptotic caspases may actually promote infection by some bacterial pathogens (Molmeret *et al.*, 2004; Srikanth *et al.*, 2010).

## Caspases – enzymatic mediators of apoptosis

Caspases, or cysteine aspartate proteases, are enzymes present in eukaryotic cells that play key roles in cellular differentiation, proliferation, inflammatory responses, and ultimately cell death (Connolly *et al.*, 2014). The enzymes are divided into pro- and non-inflammatory forms based on their ability to initiate distinctive types of cell death, with those involved in pathways such as pyroptosis (caspase-1, -4, -5, -11, -12) classed as pro-inflammatory and those involved in apoptosis (caspase-2, -3, -6 -7, -8, -9, -10) classed as being non-inflammatory (Nuñez *et al*., 1998; Lamkanfi and Dixit, 2014). While the number and type of programmed cell death (PCD) pathways is constantly evolving (as we gain a better appreciation of the complex genetic and cellular regulation of PCD), in the context of bacterial infection, the activation of distinct inflammatory or non-inflammatory PCD can have a significant effect on the induction and severity of an immune response to bacterial pathogens. Crossover and feedback/activation between inflammatory and non-inflammatory pathways occurs during infection, while neighbouring cells may also undergo distinct types of cell death in close proximity and some pathogens are known to induce differing types of cell death depending on the host cell type infected (Fink and Cookson, 2005; Rosenzweig and Chopra, 2013). Moreover, apoptotic cells have also been shown to influence their environment inducing signalling changes and cell death in neighbouring bystander cells, and inducing the extracellular release of potent caspases that can undermine epithelial integrity (Chin *et al.*, 2006; Flynn and Buret, 2008; Grant *et al.*, 2008).

## Caspase-3 – the executioner caspase

Apoptotic caspases are present in inactive pro-forms, and each is cleaved to induce its activation. Following activation of either the intrinsic or extrinsic pathways of the apoptotic cascade, initiator caspases cleave and activate the executioner caspases-3, -6 or -7. Pro-caspase-3 becomes an active enzyme when two cleaved monomers come together to form an active dimer (Nuñez *et al*., 1998); this has potent activity, including an ability to autocatalytically activate that contributes to the observed cascade effect of increasing caspase-3 activity as apoptosis progresses. Active caspase-3 recognizes a specific short peptide cleavage motif (DXXD) and cleaves cellular proteins where this motif is present and accessible (Fischer *et al.*, 2003; Ju *et al.*, 2007). The alternative executioner caspase, caspase-7, also recognizes an identical motif and has extensive functional redundancy with caspase-3 although caspase-3 is regarded as a more significant player in apoptosis and cell death because of its substrate promiscuity. (Walsh *et al.*, 2008; Lamkanfi and Kanneganti, 2010). Nevertheless, caspase-7 can also play an important role in the outcome of bacterial infection by providing an adaptive mechanism whereby the host membrane is protected from damage from pore forming toxins (Cassidy *et al.*, 2012).

The potential of caspase-3 to cause apoptosis once activated means that its activity must be tightly controlled. This control is achieved through constant turnover of the enzyme, which ensures that a threshold level of enzyme activation is not reached without an apoptotic stimulus (Tan *et al.*, 2006; Jiang *et al.*, 2009; Choi *et al.*, 2009; Lai *et al.*, 2011). Additionally, eukaryotic cells have been documented to have low levels of caspase-3 activity in non-apoptotic states implying that sub-apoptotic levels of this enzyme are expressed independent of apoptosis (Boland *et al.*, 2013; Connolly *et al.*, 2014). Of note for microbial infection, this caspase-3 activity plays roles in fundamental processes aside from apoptosis, most significantly in host cell proliferation and differentiation, but also in immunomodulation, signal transduction and cell migration. Therefore, perturbation of caspase-3 by bacterial pathogens may have consequences beyond simply deciding the fate of infected cells.

## Apoptosis and bacterial infection

Apoptosis has been established as a critical point in viral infection, acting as either a facilitator or inhibitor of viral replication (Best, 2008; Richard and Tulasne, 2012). Similarly, apoptosis was thought to be a deliberate host response to a bacterial infection that ultimately results in the removal of compromised cells. More recently, however, studies have challenged this dogma, such that apoptosis has been described as a fundamental pathway in bacterial–host interactions, but its role in inhibiting or facilitating infection is yet to be clearly defined. While apoptosis can remove infected and compromised cells to benefit the host, induction of apoptosis may carry this out in a non-inflammatory fashion while also disrupting, for example, epithelial barriers to infection or removing circulating immune cells (Grant *et al.*, 2008; Nogueira *et al.*, 2009; Peters *et al.*, 2013). This apparent paradox puts into question who benefits the most from apoptosis during infection – the bacteria or the host? While this question will remain a subject of debate, emerging evidence suggests that in some cases direct targeting of caspases is being employed by bacterial pathogens through effector proteins (Table 1). Indeed, such subversion occurs at nearly all points of the apoptotic cascade with different bacterial pathogens having evolved distinct modes to induce or inhibit specific apoptotic pathways in an attempt to manipulate the lifespan of infected cells and/or influence their behaviour in a manner that supports infection. Therefore, the critical role caspases play in determining cellular fate makes these serine proteases a high-risk target for bacterial pathogens, but when successfully manipulated, disruption of caspase function and caspase-3 in particular, can significantly undermine the host response to infection and can promote dissemination and further bacterial infection.

**Table 1:** Summary of secreted bacterial effector proteins that activate or inhibit caspase-3

Bacterium	Effect on caspase-3?	Other caspases?	Mechanism of caspase-3 activation/inhibition	Bacterial effector responsible	Consequence	Reference
*H. pylori*	Activates	Cas-8	Caspase-3 possibly activated directly	ND	Host cell killing	Ashktorab *et al.*, 2002
*L. pneumophila*	Activates	No	Caspase-3 activated directly	Dot/Icm effector	Prevents phagolysosome fusion	Molmeret *et al.*, 2004; Zhu *et al.*, 2013
*S.* Dublin	Activates	ND	ADP ribosylating activity dependent	SpvB/SPI-2 TTSS	Important pathogenic mechanism	Browne *et al.*, 2008
		No	ND but caspase-3 activated directly	PhoP regulation of *spv* locus	Survival during systemic infection	Valle and Guiney, 2005		
*S.* Typhimurium	Activates	No	Direct caspase-3 activation	SipA	Effector processing	Srikanth *et al.*, 2010
*EPEC*	Activates	ND	Cell cycle arrest activates caspase-3	Cif	Delayed apoptosis favouring colonization	Samba-Louaka *et al.*, 2009
*E. coli* O157:H7	Activates	Cas-9	Mitochondrial disruption	N-terminal of EspF	Leads to attaching/effacing lesions	Zhao *et al.*, 2013
*Y. pestis*	Activates	Cas-8	Death inducing signalling complex assembly	YopK/YopJ interplay	Alters inflammatory signalling	Peters *et al.*, 2013
*V. vulnificus*	Activates	No	Direct cleavage of caspase-3	Metalloprotease vEP	Unknown, non-specific caspase-3 activation	Kim *et al.*, 2007
*A. salmonicida*/	Activates	Cas-9	Multiple mechanisms	AexT/AexU, Act2 and Hcp	Control of host inflammatory response	Rosenzweig and Chopra, 2013
*A. hydrophila*												
*P. aeruginosa*	Activates	Cas-8	ADP-ribosylation, and possibly by caspase-3 binding	ExoS	Further dissemination	Kaufman *et al.*, 2000
*F. tularensis*	Activates	Cas-1	Cytochrome-c release	Putative effectors IglC and IglI	Further dissemination	Santic *et al.*, 2010
*S. Typhimurium*	Inhibits	ND	Sustains Akt activation	SopB	Survival of intracellular replication niche	Knodler *et al.*, 2005
*P. aeruginosa*	Inhibits	ND	Stabilizes X-IAP	Secreted protein/possibly ExoA	Inhibiting apoptosis aids bacterial survival	Ashare *et al.*, 2007
*F. tularensis*	Inhibits	ND	NF-kB induction prevents apoptosis	T6SS effector, possibly IglC	Survival of infected cells/replication	Santic *et al.*, 2010
*L. pneumophila*	Inhibits	No	Dot/Icm dependent	Dot/Icm effector	Inhibition allows time for replication	Abu-Zant *et al.*, 2007
*S. flexneri*	Inhibits	ND	Anti-apoptosis expression/direct caspase binding	MxiE secretion and effector Spa15	Protects intracellular replication niche	Clark and Maurelli, 2007; Faherty and Maurelli, 2009; Faherty *et al.*, 2010

Activation of caspase-3 by individual effectors is indicated as well as whether this activation is potentially direct or through upstream initiator or other caspases. ND indicates that the mechanism of activation of caspase-3 or the involvement of other caspases was not defined in the study.

## Bacterial effector proteins and caspase-3 activation

Bacterial secretion systems and the proteins that traverse them are essential components of the virulence arsenal of many pathogens. Many effectors are secreted through the type three secretion system, a sophisticated organelle specific to gram-negative pathogens and composed of a motor and needle complex through which secreted effectors are injected into host cells (Dean, 2011; Raymond *et al.*, 2013). The secreted effectors promote disease by co-opting host cell signal transduction pathways that facilitate cell attachment and entry, suppress the host immune/defense response, and modulate host cell biology. Consequently, these effectors play a prominent role in bacterial pathogenesis and host association. It is well appreciated that effectors constitute a large and diverse group of virulence proteins that mimic eukaryotic proteins in structure and function. In fact, up to 100 different type three secreted effector (T3SEs) proteins may be delivered into individual host cells by a single bacterium (Dean and Kenny, 2009). Moreover, T3SEs are often multifunctional proteins with many overlapping properties that orchestrate specific host cell responses, which ultimately subvert fundamental pathways linked to cell survival, inflammation and microbe destruction (Dean, 2011). Therefore, apoptosis and caspase-3 targeting by bacterial effectors is not surprising since coordinating the ability of a cell to survive or die in controlled circumstances offers obvious benefits to an invading microbe.

It appears that caspase-3 activation during bacterial infection is a common by-product of bacterial invasion, perhaps precipitated by the ensuing stress on the host cell associated with intracellular replication. An example are large molecular weight bacterial toxins that can target the cell cycle or cell integrity with the resulting off-target effect being cellular stress with subsequent cell death incited through apoptotic caspases (Heine *et al.*, 2008; Ionin *et al.*, 2008; Lee *et al.*, 2008; Cheung *et al.*, 2009). The relationship between bacterial effectors and the activation of caspase-3 is an area of increasing interest since in addition to non-specific or indirect activation of caspase-3, effectors are also able to promote caspase-3 activation through subtle changes within cellular pathways or even through direct interaction with the enzyme (Table 1). The outcome for the pathogen responsible is often an increase in infectivity rather than a clearing of the infection as expected by the conventional understanding of the protective role of apoptosis.

## *Salmonella* effectors – divide and conquer

*Salmonella* Typhimurium interactions with caspase-3 are beginning to be understood, as effectors responsible have been identified and the role that the enzyme plays in infection has been studied in detail (Takaya *et al.*, 2005; Valle and Guiney, 2005; Browne *et al.*, 2008; Srikanth *et al.*, 2010). *S*. Typhimurium uses an array of effectors to exploit host cell function in both epithelial and immune cells (McGhie *et al.*, 2009). As mentioned prior, a prominent feature shared by many effectors is their modular architecture, which is often comprised of well-defined regions that confer a subversive function. These distinct modules within an effector often mediate very different, unrelated functions, strongly suggesting that they evolved independently of each other and subsequently combined to form a chimeric protein (Kaniga *et al.*, 1996; Dean, 2011; Fookes *et al.*, 2011). This forms the basis of ‘*terminal reassortment*’, a hypothesis proposed to explain the diversity of bacterial effectors (Stavrinides *et al.*, 2006). The terminal reassortment tenet is strengthened by the finding that 32% of all type three effector families contain chimeric effectors and evidence that terminal reassortment is important for the evolution of these virulence proteins (Stavrinides *et al.*, 2006; Agbor and McCormick, 2011; Fookes *et al.*, 2011).

In keeping with this premise, we discovered that many T3SEs from *S*. Typhimurium harbour a functional caspase-3 cleavage site uniquely positioned at the junction separating their distinct functional domains, thereby producing two independently functional proteins (Srikanth *et al.*, 2010). *Salmonella* invasion protein A (SipA), is a bifunctional molecule with an actin-binding function of SipA is localized to a C-terminal fragment (Lilic *et al.*, 2003) while the N-terminal fragment triggers signal transduction cascades that promote polymorphonuclear leukocyte migration (Lee *et al.*, 2000; Wall *et al.*, 2007). SipA also harbours a functionally active caspase-3 motif that is precisely located at the junction separating the two functional domains of this protein (Srikanth *et al.*, 2010). The outcome offers a compelling explanation as to how diverse effectors with a modular architecture are able to perform multiple unrelated functions in a manner pivotal to the pathogenicity of the organism. Remarkably, SipA, itself, is necessary and sufficient for early caspase-3 activation, but in a process independent from the apoptotic cascade (Srikanth *et al.*, 2010). SipA therefore drives its own cleavage upon cell entry, a novel mechanism for activating a T3SE. Other caspase-3 cleavage sites identified in *S.* Typhimurium are also restricted to effector proteins, with no sites in type three structural proteins or chaperones, indicating this may be a general strategy employed by *S*. Typhimurium for processing of its secreted effectors.

Caspase-3 mediated proteolytic cleavage of viral effectors or capsid proteins has also been implicated in disease progression and virus spread (Zhirnov *et al.*, 1999; Wurzer *et al.*, 2003; Best, 2008; Syrtzev, 2009; Richard and Tulasne, 2012). Identified caspase-3 cleavage sites, considered to play a role in processing of viral proteins, are highly relevant in pathogenesis of influenza virus and their disruption attenuates viral virulence (Wurzer *et al.*, 2003; Zhirnov and Klenk, 2009). Similarly, in the case of *S* Typhimurium infection, a single amino acid substitution in the caspase-3 motif of SipA to a sequence not recognized by caspase-3 profoundly attenuates the virulence of this pathogen both *in vitro* and *in vivo* (Srikanth *et al.*, 2010). It is tempting to speculate that mutation of caspase-3 motifs in central virulence factors of both viral and bacterial pathogens may lead to novel vaccine approaches.

While SipA induces caspase-3 activation in intestinal epithelial cells, the SPI-2 T3SE SpvB induces caspase-3 activation in macrophages through its ADP-ribosylation of actin during infection (Table 1; Valle and Guiney, 2005; Browne *et al.*, 2008). Although the exact mechanism of caspase-3 activation remains unclear, again, it was independent of the initiator caspases-8 and -9 (Valle and Guiney, 2005). SpvB also has two functional domains but a caspase-3 site has to date not been identified but other host or bacterial proteases may be responsible. Therefore, these two T3SEs induce caspase-3 activation in different cell types through very different means, with SipA inducing caspase-3 at the earliest time point in infection (i.e. epithelial cell entry) and SpvB later in infection upon SPI-2 expression in macrophages (Valle and Guiney, 2005; Browne *et al.*, 2008; Srikanth *et al.*, 2010). It appears that during *S*. Typhimurium infection, caspase-3 is under continuous targeting by effectors.

## Caspase-3 and *Legionella* intracellular survival

*Legionella pneumophila* is thought to use effectors to directly activate caspase-3 and bypass the classical intrinsic and extrinsic pathways of apoptosis activation. One of five Dot/Icm secreted effector(s) is thought to be responsible (VipD/Lpg2831, Lpg0716, Lpg0898, Lpg1625, LegS2/Lpg2176; Zhu *et al.*, 2013). The consequences of caspase-3 activation during *L. pneumophila* infection also sheds light on one of the more diverse roles for caspase-3 in promoting infection as its activation causes degradation of rabaptin-5, a phagosome/endosome marker that marks phagosomes for lysosome fusion and bacterial killing (Zhu *et al.*, 2013). Degradation of rabaptin-5 is an essential step in mediating *L. pneumophila* intracellular replication and ensuring a successful infection. There is also speculation that multifunctional *L. pneumophila* proteins may also undergo some kind of processing post-delivery into host cells in a manner similar to *S.* Typhimurium effectors, though as yet no evidence has been presented to indicate caspase-3 may be involved (Zhu *et al.*, 2013). While direct activation of caspase-3 by bacterial effectors such as those from *L. pneumophila* and *S.* Typhimurium is intriguing in the context of infection, a greater understanding of how these effectors mediate this activation, without inducing widespread apoptosis, would be of great significance in fighting these infections. The observed temporal delays in apoptosis post-caspase-3 activation leads to the hypothesis that bacterial pathogens may employ complementary strategies for both initial caspase-3 activation and later enzyme inhibition, most likely through modification or degradation of the enzyme by other effectors to prevent rapid apoptosis.

## Extracellular release of Caspase-3 during *Escherichia coli* infection

The T3SEs Cif and EspF from *E. coli* activate caspase-3 indirectly through disruption of cellular pathways with both the cell cycle and the mitochondria being targeted (Samba-Louaka *et al.*, 2009; Zhao *et al.*, 2013). This results not only in caspase-3 activation but also its release extracellularly into the intestinal lumen during infection. Extracellular caspase-3 release has previously been described in the intestine and in the case of *E. coli* infection, it degrades tight junction proteins that are susceptible to cleavage through their caspase-3 motifs, resulting in reduced intestinal epithelial integrity (Hentze *et al.*, 2001; Bojarski *et al.*, 2004; Chin *et al.*, 2006). These proteins are integral to the integrity and barrier function of the intestinal epithelium and such caspase-3 cleavage makes the intestinal barrier vulnerable to potential bacterial paracellular translocation. The mechanism of caspase-3 mobilization and release from the cell is unknown. However, ubiquitination, which is carried out so effectively by pathogens such as *E. coli*, *Pseudomonas aeruginosa* and *S*. Typhimurium through their ubiquitin ligase mimics (i.e. NleL, AvrPtoB, SopA, SspH2), can mobilize intracellular caspase-3 (Janjusevic *et al.*, 2006; Zhang *et al.*, 2006; Quezada *et al.*, 2009; Lin *et al.*, 2011a). A similar phenomenon occurs in *S*. Typhimurium-infected epithelial cells with activated caspase-3 moving from the cytosol to the cell membrane, but again, the trigger or pathway responsible for this migration is presently unknown (Srikanth *et al.*, 2010).

## *Yersinia* spp. effectors and caspase-3

*Yersinia* spp. encodes a number of Yop proteins that manipulate pathways and caspases upstream of caspase-3 that dramatically alter its activation (Table 1). Indeed, infection by *Yersinia* spp. amounts to a coordinated attack on PCD pathways with modulation of host cell death determining the outcome of infection through alteration of the innate inflammatory response (Bergsbaken and Cookson, 2002). Activation of caspase-3 is dependent on the interplay between Yop proteins in the case of *Y. pestis*, with YopK influencing the induction of caspase-3 activation by the deubiquitinase YopJ (Peters *et al.*, 2013). While YopJ induces apoptosis through inhibiting the production of anti-apoptotic proteins, YopK further manipulates apoptosis by altering the role upstream caspases play in activating caspase-3. Perhaps most interestingly, while YopK controls YopJ translocation, its deletion had differing effects on YopJ induction of apoptosis depending on the cell type infected. Excess YopJ was expected to induce increased caspase-3 activation in infected cells but this occurs at varying levels in different macrophage cell lines leading to speculation that activation of caspase-3 by these Yop proteins may be related to the activation state of infected immune cells (Peters *et al.*, 2013).

The outcome is that host cells infected by *Yersinia* spp. are driven towards non-inflammatory apoptosis through caspase-3 activation, reducing the influx of immune cells and increasing the likelihood of bacterial survival and dissemination. Again, like for many other pathogens discussed in this review, caspase-3 activation is most likely dependent on the cell type infected, and in the case of immune cells, their activation state (Bergsbaken and Cookson, 2002; Peters *et al.*, 2013). Whether this increased complexity of interplay between caspase-3 and effectors in different cell types is due to differing host cell responses to infection or an adaptation by bacterial pathogens to their environment is still to be elucidated (Rosenzweig and Chopra, 2013).

## *Vibrio* metalloprotease *Vibrio* extracellular protease (vEP) cleaves caspase-3

*Vibrio vulnificus* has perhaps one of the most intriguing mechanisms of caspase-3 activation, achieved through its secreted metalloprotease vEP. This small secreted enzyme not only directly activates caspase-3 but does so in a unique way, cleaving the enzyme at a site distinct from the normal cleavage motif targeted by initiator caspases to activate the enzyme (Kim *et al.*, 2007). This novel mechanism of vEP cleavage of caspase-3 causes the enzymes activity to initially and profoundly increase before more cleavage of caspase-3 by vEP renders the enzyme inactive at later time points in infection. Such vEP activity is non-specific, and although vEP is seen intracellularly during infection, it has yet to be shown to be responsible for induction of apoptosis. However, such transient activation implies that caspase-3 activity would only present at early stages in infection, and is similar to that seen in other bacterial infections where an initial increase in caspase-3 activity is followed by a delay in triggering apoptosis thought to be as a result of the intervention of other anti-apoptotic effectors (Srikanth *et al.*, 2010). This is yet another example in which diverse bacterial pathogens are using contrasting means to achieve similar goals, with *V. vulnificus* employing a single enzyme to control caspase-3 activity whereas other bacterial pathogens may use a number of effectors to achieve the same goal (Kim *et al.*, 2007).

## *Aeromonas* effectors and caspase-3

*Aeromonas salmonicida* and *A. hydrophila* employ a number of effectors that activate caspase-3 (Table 1). Three effectors have been implicated; AexT/AexU, Act2 and Hcp. AexT from *A. salmonicida* is a bifunctional effector protein, homologous to ExoT/S from *Pseudomonas*, which is capable of caspase-3 activation through induction of caspase-9 activity (Rosenzweig and Chopra, 2013). When its homologue from *A. hydrophila*, AexU, is mutated, mutants are far more virulent resulting in increased cytokine production and mouse mortality during infection. AexU therefore is speculated to play an important role in inducing apoptosis as a means of controlling the host inflammatory response and prolonging *A. hydrophila* infection. The effector Act2 also induces caspase-3 activation and apoptosis but the mechanisms are incompletely understood while the effector Hcp, once translocated into the host cell, induces rapid caspase-3 activation (Rosenzweig and Chopra, 2013). Macrophages treated with Hcp also lose the ability to carry out phagocytosis indicating this may be a means of protection and escape from infected immune cells. Multiple functional copies of Hcp are present on *Aeromonas* genomes and these can be expressed simultaneously allowing rapid induction of apoptosis, emphasizing the important role that manipulation of host life span plays during infection by this *Aeromonas*.

## *Francisella*, *Pseudomonas* and caspase-3 activation

Other effectors known to target caspase-3 indirectly include ExoS from *P. aeruginosa* and type six secretion system (T6SS) delivered effectors from *Francisella tularensis* (Lai and Sjöstedt, 2003; Alaoui-El-Azher *et al.*, 2006; Jansson *et al.*, 2006; Santic *et al.*, 2010; Zivna *et al.*, 2010). *P. aeruginosa* ExoS, a bifunctional homologue of AexT/AexU from *Aeromonas*, inhibits phosphorylation of cellular proteins such as FOXO3a, inducing caspase-3 activation and the apoptotic cascade (Jansson *et al.*, 2006). Interestingly, in the case of *P. aeruginosa*, caspase-3 activation by ExoS was noted earlier than that of the upstream initiator caspase, caspase-8, suggesting that ExoS may directly activate caspase-3, in addition to indirectly activating the enzyme through caspase-8 (Kaufman *et al.*, 2000). *F. tularensis* induction of caspase-3 is dependent on a functional T6SS, leading to cytochrome-C release and nuclear-factor kappaβ (NF-κβ) translocation (Santic *et al.*, 2010). This effect depends on IglC and IglI, putative *Francisella* effectors that also play a structural role in the T6SS. For both *P. aeruginosa* and *F. tularensis*, caspase-3 activation and apoptosis appears to result in the death of host cells with subsequent bacterial dissemination. This is yet another example in which the role of apoptosis rather than combating infection contributes to infection by aiding bacterial spread.

## Mechanisms of caspase-3 activation

Activation of caspase-3 by bacterial pathogens is increasingly being recognized as a bacterial infection strategy but yet little is known of the mechanisms by which bacteria interact with caspase-3 directly. Upstream initiator caspases such as caspase-8 and -9 are routinely activated during infection resulting from the perturbation of numerous cellular pathways, often simultaneously, leading to indirect induction of caspase-3 activity and the cell being overwhelmed by the pathogen. While this activation can be tracked over time giving an indication of the pathways involved and how caspases are activated, direct or alternative activation of caspase-3 as described during bacterial infection is more difficult to understand mechanistically (Kaufman *et al.*, 2000; Kim *et al.*, 2007; Srikanth *et al.*, 2010; Zhu *et al.*, 2013). Intrinsic and extrinsic apoptotic pathways are thought to be the means by which the majority of apoptosis occurs but activation of caspase-3 independently of these pathways suggests an alternative pathway(s) for caspase-3 activation is induced during bacterial infection. As such, a pathway has yet to be identified; it cannot be discounted that direct effector enzyme interaction may also be responsible for caspase-3 activation. Direct effector binding of caspases, as shown with *E. coli* NleF (caspases-4, -8 and -9), *Shigella flexneri* OspC3 (cleaved caspase-4) and YopM of *Y. pestis* (caspase-1), may also occur with caspase-3, although to date, this remains largely speculation (LaRock and Cookson, 2012; Blasche *et al.*, 2013; Kobayashi *et al.*, 2013). The presence within effectors of short amino acid motifs that are known to stimulate caspase-3 activation, such as the RGD motif, may also contribute to activation, and indeed, an evolutionary conservation of a prokaryotic caspase-3 activity has also been described, which could play a role (Buckley *et al.*, 1999; Bidle *et al.*, 2010). However if, as the evidence suggests, some bacterial pathogens do indeed utilize unique means of targeting such a critical host enzyme, the findings would have wide reaching repercussions outside bacterial infection (Kim *et al.*, 2007; Srikanth *et al.*, 2010).

In the future, detection of subtle modifications or manipulations of caspase-3 may be crucial to furthering our understanding of host–pathogen interactions given that many bacterial pathogens may use strategies of enzyme manipulation rather than inducing its transcriptional (up or down) regulation. Indeed, in the case of *S*. Typhimurium infection, there is no increase in caspase-3 mRNA levels upon infection, indicating that rather than increased production of caspase-3 in infected cells, the enzyme that is present in the cell at low levels pre-infection is activated and mobilized upon bacterial invasion (Srikanth *et al.*, 2010). The presence of both active and pro-, or inactive, forms of caspase enzymes within host cells add an extra layer of complexity especially as fine-tuned modifications of caspases by small molecules can prevent their activation or inhibit their activity.

## Inhibition of caspase-3 by bacterial pathogens

Intracellular survival of bacterial pathogens determines the success or failure of an infection and bacterial pathogens have evolved to protect their intracellular niche to increase their chances of success. While activation of apoptosis-related proteins, as described above, appears an unusual strategy and detrimental to intracellular survival, many bacterial pathogens actively engage in complementary strategies to inhibit apoptosis (Faherty and Maurelli, 2008). *F. tularensis*, *L. pneumophila*, *P. aeruginosa* and *S*. Typhimurium all employ effectors to manipulate caspase-3 activation, but in this case, in order to inhibit its activity (Knodler *et al.*, 2005; Abu-Zant *et al.*, 2007; Ashare *et al.*, 2007; Santic *et al.*, 2010). Each, however, affects caspase-3 indirectly, primarily through activation of pathways or proteins that prevent caspase-3 activity such as NF-κβ, inhibitor of apoptosis protein (IAP) or Akt. Inhibition by both *L. pneumophila* and *F. tularensis* is dependent on their Dot/Icm and T6SS (Abu-Zant *et al.*, 2007; Santic *et al.*, 2010). Although the effectors responsible for manipulating caspase-3 activity are not definitively known, IglC is suggested as being responsible in the case of *F. tularensis*, The outcome is similar for both pathogens with NF-κβ translocated to the nucleus where it increases expression of anti-apoptotic proteins. The net result for these pathogens is increased replication time in their intracellular niche and protection from circulating immune cells.

*P. aeruginosa* and *S*. Typhimurium both target/phosphorylate and stabilize Akt reducing caspase-3 activation while *P. aeruginosa* also stabilizes X-linked IAP, preventing activation of apoptotic caspases (Knodler *et al.*, 2005; Ashare *et al.*, 2007). The strategy of stabilizing anti-apoptotic proteins is a common approach for inhibiting caspase-3 utilized by bacterial pathogens. *S. flexneri* uses a combination of these approaches, including up-regulating the IAP family (Faherty *et al.*, 2010). In addition, *S. flexneri* inhibits caspase-3 through a membrane expression of invasion plasmid antigen E (MxiE)-dependent mechanism that may involve direct binding of caspase-3 or caspase-9 (Clark and Maurelli, 2007). The effector responsible is not yet known but Spa15 is known to have an anti-apoptotic effect (Faherty and Maurelli, 2009). Enteropathogenic *E. coli* (EPEC) NleH, a homologue of the *Shigella* effector OspG, also inhibits caspase-3 activation through interaction with Bax inhibitor 1 (Hemrajani *et al.*, 2010). In the case of *Shigella* and *E. coli* infections, this inhibition of caspase-3 activation and subsequent apoptosis may slow exfoliation of the intestinal epithelium and promote infection by these pathogens.

## Mechanisms of inhibition

Recent studies have shown that effector binding of caspases other than caspase-3 can have an inhibitory effect on enzyme activity (Blasche *et al.*, 2013). While direct binding can inhibit caspase enzyme activity, subtle caspase-3 modifications can also have a significant effect on enzyme activity, intracellular location and its lifespan (Choi *et al.*, 2009; Jiang *et al.*, 2009; Dunne *et al.*, 2013). Bacteria not only possess the effectors to inhibit caspases in this way but pathways such as ubiquitination and S-nitrosylation are up-regulated in infected host cells (Janjusevic *et al.*, 2006; Zhang *et al.*, 2006; Quezada *et al.*, 2009; Lin *et al.*, 2011b; Dunne *et al.*, 2013). Ubiquitination of caspase-3 reduces activity of caspase-3 by altering the active site while also targeting the enzyme for proteasomal degradation. This mechanism of proteasomal recycling is employed by host cells to maintain caspase-3 at basal non-apoptotic levels at times when no danger is perceived (Tan *et al.*, 2006). The presence of large numbers of ubiquitin ligase mimics in the bacterial effector repertoire means ubiqutination may be a means for bacterial pathogens to secure their intracellular niche for prolonged periods, and also could explain the mobilization of caspase-3 to the extremities of the cell seen during infection with *S*. Typhimurium and *E. coli* (Flynn and Buret, 2008; Srikanth *et al.*, 2010).

An alternative means of caspase-3 inhibition recently identified in long-term *E. coli* infection of immune cells, although not yet attributed to a specific effector or pathway, was S-nitrosylation (Dunne *et al.*, 2013). This modification occurs in all host cell types but was seen to be up-regulated in infected dendritic cells and macrophages leading to both increased proteasomal degradation of caspase-3 and insertion of S-nitrosyl groups in the enzyme active site, reducing or eliminating its enzymatic activity. The result was prolonged survival for infected cells. S-nitrosylation of caspase-3 was not observed in infected epithelial cells again highlighting the difference in response of differing cell types as regards caspase-3 activation (Dunne *et al.*, 2013). S-nitrosylation is also employed by bacteria to control protein stability and homologous proteins to those in host cells are used to mediate the transfer of S-nitrosyl groups to targeted proteins (Mitchell and Marletta, 2005; Gusarov and Nudler, 2012; Seth *et al.*, 2012). Direct S-nitrosylation of host proteins to date has not been demonstrated but perturbation of host cell pathways by bacteria may be responsible for alterations in host cell S-nitrosylation pathways.

## Future directions to understand caspase-3 manipulation

The essential role of caspase-3 in apoptosis has caused it to become the focus of much attention over the last 40 years. Similarly, its targeting by bacterial and viral pathogens has increased the attention on the role caspase-3 plays in enabling pathogens to survive intracellularly or induce death of targeted cells. The discovery that caspase-3 has functions independent of apoptosis such as immune signalling, cell differentiation and cell migration support the notion that the bacterial effector–caspase-3 interactions discussed herein are having effects far beyond those simply related to apoptosis. Effectors that target caspase-3 will likely, over the lifespan of the infected cell, affect the role that cell plays through altering its immune signalling, localization and ability to continue its cell cycle. These disturbed cells can also have dramatic and detrimental effects on neighbouring cells, inducing their death and potentially leading to breaches in integrity of host barriers or the host immune response. Future efforts to understand how bacteria use their effectors to manipulate caspase-3 should not only shed light on the outcome of infection but also how caspase-3 controls essential pathways outside apoptosis.
